# Connectivity Changes Underlying Neurofeedback Training of Visual Cortex Activity

**DOI:** 10.1371/journal.pone.0091090

**Published:** 2014-03-07

**Authors:** Frank Scharnowski, Maria Joao Rosa, Narly Golestani, Chloe Hutton, Oliver Josephs, Nikolaus Weiskopf, Geraint Rees

**Affiliations:** 1 Wellcome Trust Centre for Neuroimaging, UCL Institute of Neurology, University College London, London, United Kingdom; 2 UCL Institute of Cognitive Neuroscience, University College London, London, United Kingdom; 3 Institute of Bioengineering, Swiss Institute of Technology Lausanne (EPFL), Lausanne, Switzerland; 4 Department of Radiology and Medical Informatics – CIBM, University of Geneva, Geneva, Switzerland; 5 Department of Neuroscience, Institute of Psychiatry, King’s College London, London, United Kingdom; 6 University Medical School, University of Geneva, Geneva, Switzerland; Human Brain Research Center, Japan

## Abstract

Neurofeedback based on real-time functional magnetic resonance imaging (fMRI) is a new approach that allows training of voluntary control over regionally specific brain activity. However, the neural basis of successful neurofeedback learning remains poorly understood. Here, we assessed changes in effective brain connectivity associated with neurofeedback training of visual cortex activity. Using dynamic causal modeling (DCM), we found that training participants to increase visual cortex activity was associated with increased effective connectivity between the visual cortex and the superior parietal lobe. Specifically, participants who learned to control activity in their visual cortex showed increased top-down control of the superior parietal lobe over the visual cortex, and at the same time reduced bottom-up processing. These results are consistent with efficient employment of top-down visual attention and imagery, which were the cognitive strategies used by participants to increase their visual cortex activity.

## Introduction

Successful visual perception depends on the interplay in visual cortex between ongoing spontaneous activity and that evoked by a stimulus [1], [2], [3], [4], [5]. While the latter is mainly determined by stimulus characteristics, the former can be modulated by general factors including visual-spatial attention [6], [7]. Alternatively, real-time functional magnetic resonance imaging (fMRI) neurofeedback has recently been used to modulate regionally specific spontaneous brain activity [8], [9], [10]. In the field of vision, two studies applied this new method in order to train participants to voluntarily control the level of ongoing spontaneous activity in visual cortex activity [11], [12]. Both studies showed that after successful training, perception improved when participants voluntarily increased activity in a circumscribed region of their early visual cortex.

However, the mechanisms underlying neurofeedback learning are still unresolved [8], [13]. Learning voluntary control over activity within a region of interest (ROI) can induce network changes [14], [15], [16], [17]. Consistent with this, in our earlier study we found that learning control over early visual cortex activity correlated with increased functional connectivity between the visual target ROI and the superior parietal lobe contralateral to the visual target ROI (cSPL) [12]. The SPL is involved in directing covert visual-spatial attention and cognitive control [18], [19], [20], [21], [22], [23] and the increase in functional connectivity between the visual ROI and the cSPL with training might thus be a correlate of increasing attentional and cognitive control to learn self-regulation.

These results were obtained using an exploratory psychophysiological interaction analysis (PPI), which is a data-driven measure of effective connectivity. In general, a PPI analysis allows for identifying correlations between haemodynamic time series measured in different brain areas, and whether they changed depending on a psychological task [24]. In our experiment, the PPI analysis was used to identify brain areas whose connectivity to the visual target ROI changed depending on whether participants were up-regulating or not. However, PPI has three important limitations: (1) PPI does not allow inferences about the directionality of any connectivity because it only identifies correlations between haemodynamic signals, (2) PPI is a static model that ignores time-series properties of the data, and (3) the causal interpretability of PPI is limited because it operates at the level of the blood-oxygen-level-dependent (BOLD) signal rather than on the neuronal level [25].

Here, we overcame these limitations by re-investigating the neural underpinnings of successful self-regulation in our previous study but now using dynamic causal modeling (DCM) [26], [27], [28], [29], [30]. DCM is a measure of effective connectivity that allows for investigating how brain areas interact during different experimental conditions. In contrast to PPI, DCM (1) allows for determining directionality of connectivity, (2) describes how neural dynamics propagate through a network, and (3) allows for modeling effective connectivity at the neuronal level. DCM is a model-based approach that makes use of prior knowledge about the ROIs involved, about the connections between these ROIs, and about the context dependent manipulations of the network. Based on the results of our previous exploratory PPI analysis [12], we focused the DCM analysis on characterizing effective connectivity changes between the (trained) visual ROI and the cSPL. We hypothesized that neurofeedback training leading to up-regulation of the visual ROI was mediated by increased top-down effective connectivity from the cSPL to the visual ROI that evolved with training. Further, we hypothesized that these changes would be specific to those participants who learned self-regulation of the visual ROI (the learners), i.e. participants who did not learn control over their visual ROI (the non-learners), and participants who received sham feedback (the controls) and therefore also did not learn self-regulation will not show such connectivity changes.

## Materials and Methods

Details about the data acquisition, the participants, and the neurofeedback training can be found in [12]. For completeness, the main parameters are repeated here.

### Ethics Statement

The research was conducted in accordance with the Declaration of Helsinki, and all participants gave written informed consent prior to participating in the experiment. The study was approved by the ethics committee of the Joint NHS National Research Ethics Service of the National Hospital for Neurology and Neurosurgery & the Institute of Neurology, UK.

### fMRI Data Acquisition

All experiments were performed on a 3T Magnetom Allegra head only scanner, using a standard transmit-receive head coil (Siemens Healthcare, Erlangen, Germany). Functional data were acquired with a single-shot gradient echo planar imaging sequence (matrix size: 64×64; field of view: 192×192 mm; isotropic resolution: 3×3×3 mm; 32 slices with ascending acquisition; slice thickness: 2 mm; slice gap: 1mm; echo time TE: 30 ms; TR: 1920 ms; flip angle: 90°; receiver bandwidth: 3551 Hz/Px). In the middle of each scanning session, double-echo FLASH fieldmaps (TE1∶10 ms; TE2∶12.46 ms; resolution: 3×3×2 mm; slice gap: 1 mm) were acquired and used to correct geometric distortions in the images due to field inhomogeneities.

The neurofeedback setup used Turbo-BrainVoyager (Brain Innovation, Maastricht, The Netherlands), custom real-time image export tools programmed in ICE VA25 (Siemens Healthcare) [31], and custom scripts running in MATLAB (Mathworks Inc., Natick, MA, USA). This allowed participants to be shown visual representations of BOLD signal changes in specific brain regions (in the form of a thermometer display projected into the scanner) with a delay of less than 2 s from the acquisition of the image. Head motion was corrected in real-time using Turbo-BrainVoyager. Heart rate and respiration were continuously monitored throughout the experiment (setup similar to [32]).

### Participants

**Figure 1:**
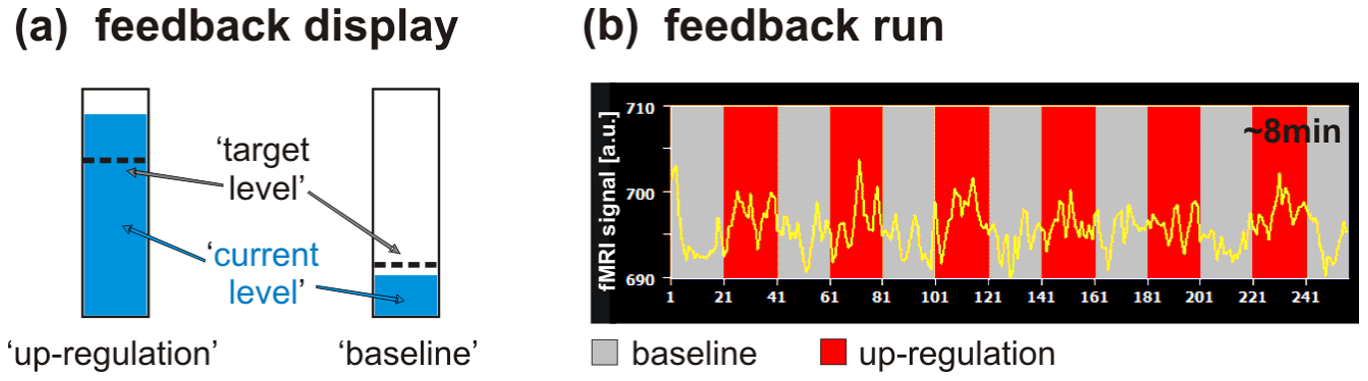
Feedback display and feedback run. (a) Custom-made software was used to continuously provide visual feedback of local brain activation to the participant in the scanner. The neurofeedback display consisted of a thermometer, and the temperature reading indicated the current level of activity in the visual cortex ROI. A dashed line indicated the target activation level, which could either be high (up-regulation condition) or low (baseline condition). (b) In each neurofeedback training session, volunteers participated in an average of ∼2 feedback runs of 8.3 min each. A feedback run was composed of 38 s baseline blocks (grey) interleaved with 38 s up-regulation blocks (red).

Sixteen naïve human volunteers (6 male, ages between 18 and 37 years, all right handed) with normal or corrected-to normal vision took part in the study. Before the experiment, they received written instructions describing that they will learn to regulate their visual cortex activity with the help of neurofeedback. The instructions included an explanation of the neurofeedback thermometer display (Figure 1a) and recommended as potential regulation strategies the use of visual imagery with high resolution details as well as changing stimulus quality (color, shape) and intensity (brightness) spatially overlapping with the target ROI. We also suggested that participants prepare a few imagined patterns in advance and to try them repeatedly. It was emphasized that participants should find an individual strategy that worked best for them. Further, they were instructed to fixate on the central fixation point throughout the experiment, to breathe steadily, and to remain as still as possible. After each scanning session, participants were asked to fill in a written questionnaire and amongst other questions, describe how they tried to manipulate the feedback signal (including drawing any visual imagery), how effective their strategy was, and how they rated the attentional demands.

### Delineation of the Visual Target ROI

In a separate scanning session before the neurofeedback training, we collected from each participant a high resolution T1-weighted structural scan of the whole brain (3D MDEFT; 1 mm isotropic resolution; matrix size: 256×240 mm; field of view: 256×240 mm; 176 sagittal partitions; echo time 2.4 ms; repetition time: 7.92 ms; inversion time: 910 ms; flip angle: 15°; readout bandwidth: 195 Hz/pixel; spin tagging in the neck with flip angle 160° in order to avoid flow artifacts) for superposition of functional maps [33]. In this first session, we also determined the visual target ROI from which participants received neurofeedback by acquiring 2 functional localizer runs of 150 volumes each. The visual localizer consisted of a flickering circular checkerboard (100% contrast, 10 Hz contrast reversal) with a diameter of 2° visual angle which was presented for ∼13 s in each of the four quadrants of the visual field (eccentricity: 3° visual angle), with a baseline condition of the same duration once after the stimulus had been presented in each quadrant. Participants received feedback from the visual ROI corresponding to active voxels in occipital cortex that responded to stimulation in the lower left or the lower right visual field (randomly assigned). The target ROI for the control group, i.e. the ventral striatum, was anatomically defined using Brain Voyager QX (Brain Innovation, Maastricht, The Netherlands).

### Neurofeedback Training

Participants took part in at least three neurofeedback training sessions spread over the course of several days. The same ROI was targeted in all training sessions. For each training session, participants performed on average 2 training runs of 8.3 min each. The training runs were composed of seven 38 s baseline blocks interleaved with up-regulation blocks of the same duration (Figure 1b). During the baseline blocks the target level indicator of the thermometer display was low, which indicated to the participants that they should mentally count backwards from 99 in steps of −7 in order to maintain a stable baseline activity (Figure 1a). During the up-regulation blocks, the target level indicator moved up, which indicated to the participants that they should increase activity in their visual ROI. Participants were presented feedback about their success via the thermometer reading, which indicated the percentage of signal change compared to the previous baseline block. With the help of the feedback, participants attempted to learn, by trial and error and using a freely chosen strategy, to up-regulate the activity in their visual ROI to the target level. No other visual stimuli were presented.

### Offline Data Pre-processing

Offline data analysis used SPM8 (Wellcome Trust Centre for Neuroimaging, Queen Square, London, UK; http://www.fil.ion.ucl.ac.uk/). The first 3 volumes of each run were excluded from statistical analysis since it takes a few volumes for T1-related equilibration to occur at the start of each fMRI run. The remaining images were corrected for slice time acquisition differences, realigned to the first scan of each run, corrected for static magnetic field (B0) inhomogeneities [34], coregistered to the structural scan and smoothed with an isotropic Gaussian kernel with 4 mm full-width-at-half-maximum (FWHM). Functional images were normalized to the MNI standard template using DARTEL [35]. Images of those participants whose visual target ROI was located in the left hemisphere were flipped so that all visual target ROIs were displayed on the right side.

### DCM Analysis

For our analysis, we used DCM 10 as implemented in SPM 8. Due to the inter-participant variability in our study and in order to generalize the results to the population, we used a random effect (RFX) Bayesian model selection approach for our DCM analysis [36]. We used a hierarchical approach, in that we first applied family-level inference procedures to investigate which general model structure underlay successful up-regulation. Subsequently, we used parameter-level inference procedures to investigate which changes in connectivity strength mediated learning to up-regulate the visual cortex. The analysis was carried out separately for the three experimental groups, i.e., the learners (N = 7), the non-learners (N = 4), and the controls (N = 5).

#### Model space

Based on the results from our previous study, we considered 2 ROIs for our DCM analysis: the visual ROI and the cSPL. The visual ROI corresponded to the individually localized ROI from which the respective participant received neurofeedback. The cSPL was based on the group result from the previous PPI analysis (MNI coordinates: (22, −58, 63), [12]). For each participant, the time courses for the visual ROI and for the cSPL were extracted, and detrended with linear and quadratic terms. Due to the small number of nodes (visual ROI, cSPL) and having only one external input (up-regulation), we did not have to limit our model space and took all possible connectivity architectures into account.

#### Avoiding double dipping or circularity in the analysis

Whereas the visual ROIs were defined with separate functional localizer runs, the cSPL ROI was defined based on data from the last neurofeedback training run. Specifically, it was defined based on a voxelwise one-sample t-test of the PPI interaction term contrast image of each learner’s last training run. The data of the DCM analyses from the non-learners, from the controls, and from all but the last training run of the learners is therefore independent of the ROI selection, thus avoiding circularity [37]. However, an analysis of the last training run based on models containing the cSPL ROI might result in circularity of the analysis. We therefore ran the same analyses excluding the last run, making these analyses independent of the cSPL ROI selection. Because the results were similar, we report here the results for the complete neurofeedback training data (including the last training run), but also report the results for the data excluding the last training run.

#### Family-level inference

**Figure 2:**
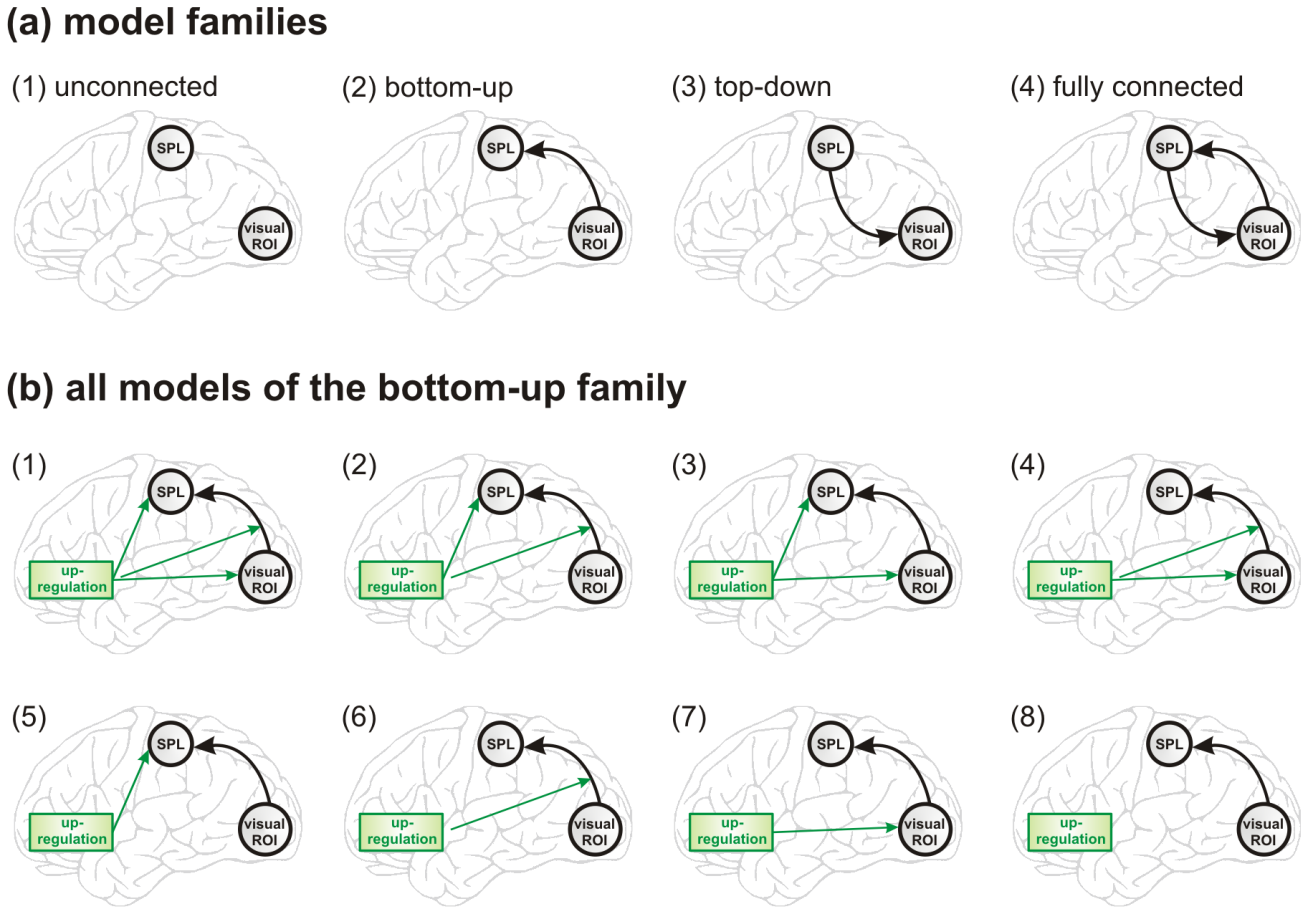
Model space partitioning. (a) For family-level inference the model space was partitioned into 4 subsets with different patterns of connectivity. (1) No connection between the visual ROI and the cSPL, (2) a bottom-up connection from the visual ROI to the cSPL, (3) a top-down connection from the cSPL to the visual ROI, and (4) a bottom-up as well as a top-down connection between the visual ROI and the cSPL. (b) As an example, the bottom-up model family contained 8 different models, which differed in how up-regulation affects the network. Up-regulation can affect both ROIs, either of the ROIs, or no ROI, and it can affect the bottom-up connection between the visual ROI and the cSPL.

To investigate which general model structure underlay successful up-regulation, we partitioned the model space in subsets of four model families that differed in the connectivity pattern between the visual ROI and the cSPL. The first family contained all models where there was no connection between the visual ROI and the cSPL (4 models), the second family contained all models where there was a bottom-up connection from the visual ROI and the cSPL (8 models), the third family contained all models where there was a top-down connection from the cSPL to the visual ROI (8 models), and the fourth family contained all models where there was a bottom-up as well as a top-down connection between the visual ROI and the cSPL (16 models) (Figure 2a). As an example, all models of the bottom-up family are illustrated in Figure 2b. Using Bayesian model selection (BMS), information over models in each model family was pooled and compared collectively [38]. Results were reported as expected and as exceedance family probabilities separately for each experimental group. A probability higher than 0.25 indicated dominance of one particular model family compared to the other model families. To assess changes across neurofeedback training, the analysis was carried out separately for the first and for the last training run.

#### Parameter-level inference

**Figure 3:**
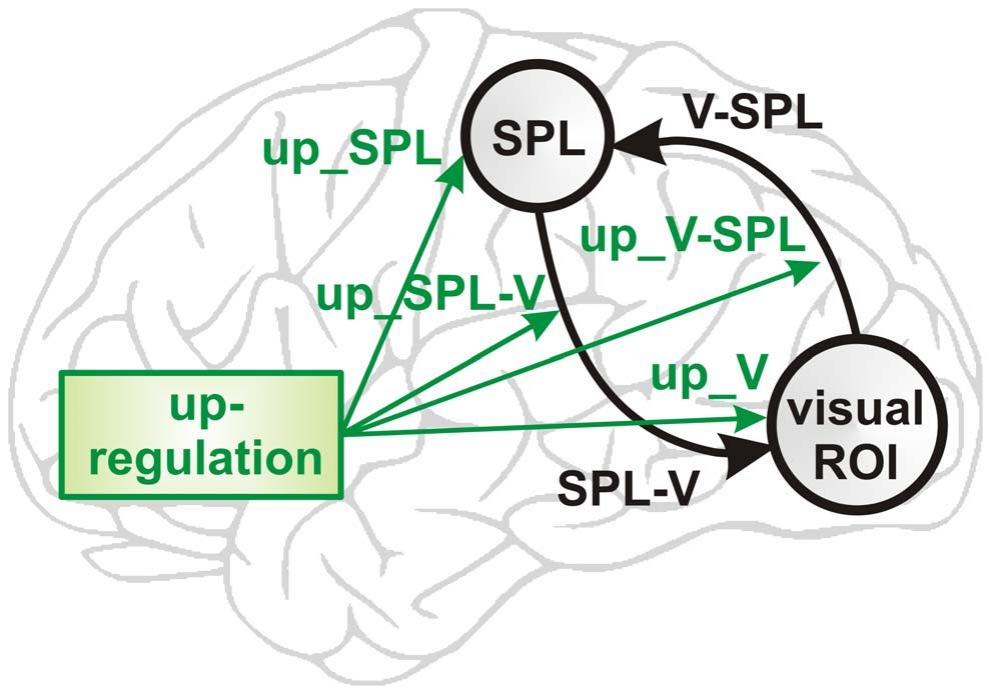
BMA parameters. Based on the fully connected model, 6 BMA parameters were investigated: The bottom-up connection strength from the visual ROI to the cSPL (V-SPL), the top-down connection strength from the cSPL to the visual ROI (SPL-V), the effect of up-regulation on the visual ROI (up_V), on the cSPL (up_SPL), on the bottom-up connection strength (up_V-SPL), and on the top-down connection strength (up_SPL-V).

Having identified the fully-connected model family as the most likely model architecture (see Results, below), we then investigated the parameters of the models within that family. The parameters of interest were the bottom-up connection strength (effective connectivity) from the visual ROI to the cSPL (V-SPL), the top-down connection strength from the cSPL to the visual ROI (SPL-V), the effect of up-regulation on the visual ROI (up_V), on the cSPL (up_SPL), on the bottom-up connection strength (up_V-SPL), and on the top-down connection strength (up_SPL-V) (Figure 3).

Because the optimal model differed between the experimental groups, we applied Bayesian model averaging (BMA) to the 16 models comprising the fully connected model family. BMA computes a weighted average of each model parameter within the model family, where the weighting depends on the evidence for each of the contributing models, i.e., the posterior probability [38], [39]. In order to compare between the experimental groups, BMA was applied separately for each group. In order to assess changes across neurofeedback training runs, BMA was applied separately for each of the 6 neurofeedback training runs.

For statistical analyses of the BMA parameters, we calculated a 3×2×6 mixed analysis of variance (ANOVA) with between-subjects factors group (learners, non-learners, or controls), and within-subjects factors training run (first run, last run) and BMA parameters (V-SPL, SPL-V, up_V, up_SPL, up_V-SPL, up_SPL-V). Due to a strong 3-way interaction trend, and due to the predicted differential performance of the BMA parameters after compared to before training across the three groups, we performed 2-way repeated measures ANOVAs, with the factors training run and BMA parameter in the 3 groups separately. To better characterize connection strength increases or decreases across all 6 neurofeedback training runs in the learners, we calculated linear regressions of each BMA parameter across runs. The statistical significance was thresholded at p<0.05.

## Results

As reported previously, 7 participants successfully learned to control activity in their visual ROI [12]. Specifically, these individuals showed a significant BOLD signal increase in the visual ROI associated with training and a significant difference in signals comparing blocks in which they were asked to increase the level of ongoing BOLD signals in the visual target ROI with baseline blocks in which active control was not exerted. Four participants did not learn to increase visual cortex activity, although they did not differ from the learners with respect to the composition of the visual ROI, the size of the ROI, the amount of training, the mental strategies used, their attentional efforts, or their vividness of visual imagery. Participants in the control group were provided with the same instructions and underwent the identical training procedure but received feedback from an area not involved in visual processing, i.e., the ventral striatum. Participants in this group did not learn to control visual cortex activity.

### Family-level Inference

**Figure 4:**
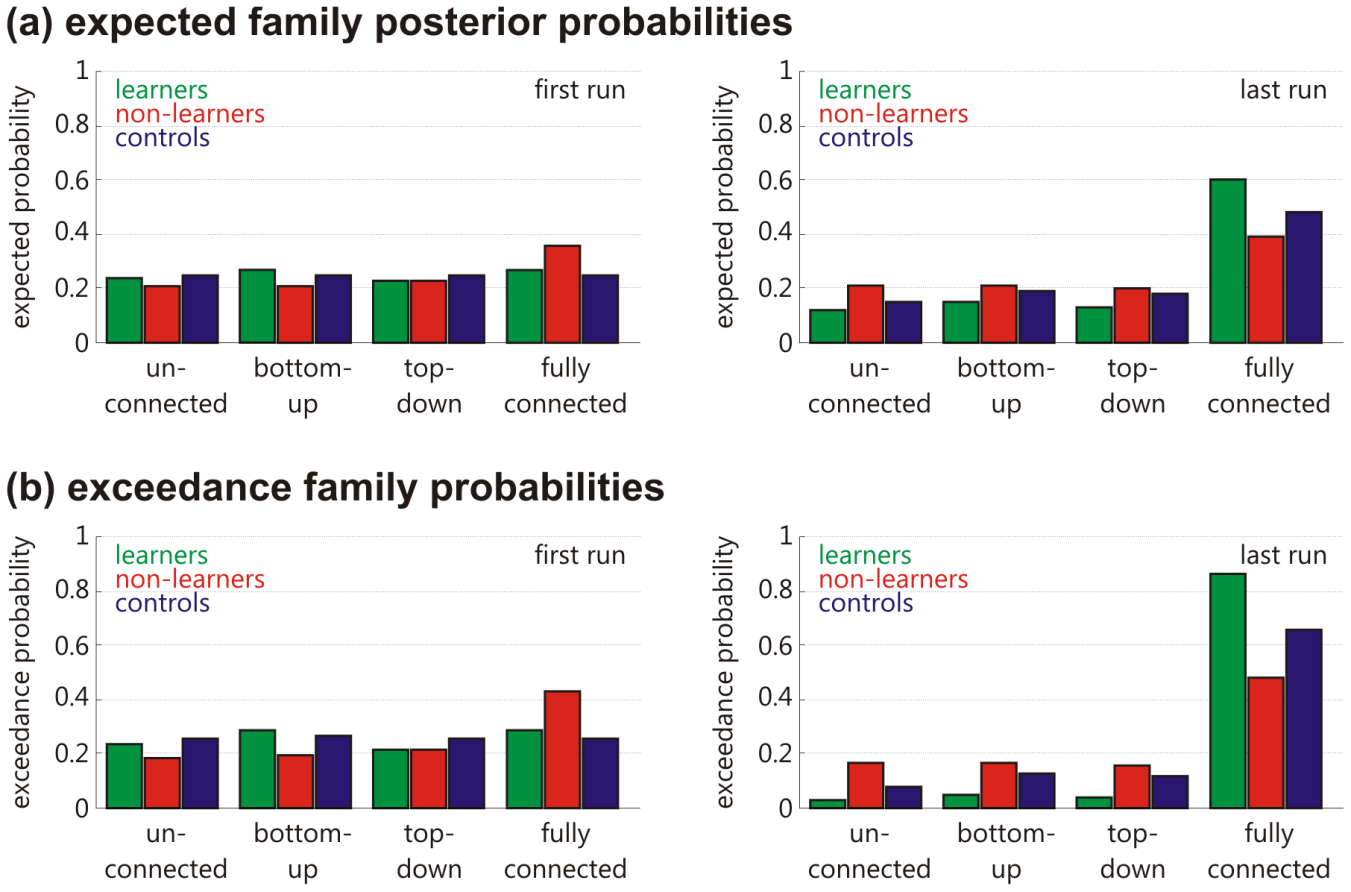
Estimated family-level probabilities. Both, the (a) expected family posterior probabilities as well as the (b) exceedance family probabilities did not show a clearly dominant model family in the first neurofeedback training run. For the last training run, the fully connected model family was more likely than the other model families, and this in all experimental groups.

An analysis of the estimated expected family posterior probabilities and the exceedance family posterior probabilities revealed that for the first neurofeedback training run, none of the model families dominated in any of the experimental groups (Figure 4; Table 1). However, in the last neurofeedback training run, the fully connected model family clearly dominated. For the learners, the exceedance probability of the fully connected model reached 0.88, for the non-learners 0.49, and for the controls 0.67. Hence, there is strong evidence that the fully connected model is the best model architecture to explain the data in the last training run, and this for all experimental groups.

**Table 1:** Family-level inference.

Expected family posterior probabilities: first training run				
experimental group	model families			
	not connected	bottom-up	top-down	fully connected
learners	0.24	0.27	0.23	0.27
non-learners	0.21	0.21	0.23	0.36
Controls	0.25	0.25	0.25	0.25
**Expected family posterior probabilities: last (second to last) training run**				
**experimental group**	**model families**			
	**not connected**	**bottom-up**	**top-down**	**fully connected**
learners	0.12 (0.14)	0.15 (0.20)	0.13 (0.30)	0.60 (0.35)
non-learners	0.21 (0.18)	0.21 (0.19)	0.20 (0.22)	0.39 (0.41)
Controls	0.15 (0.25)	0.19 (0.26)	0.18 (0.25)	0.48 (0.24)
**Exceedance family probabilities: first training run**				
**experimental group**	**model families**			
	**not connected**	**bottom-up**	**top-down**	**fully connected**
learners	0.23	0.28	0.21	0.28
non-learners	0.18	0.19	0.21	0.42
Controls	0.25	0.26	0.25	0.25
**Exceedance family probabilities: last (second to last) training run**				
**experimental group**	**model families**			
	**not connected**	**bottom-up**	**top-down**	**fully connected**
learners	0.03 (0.06)	0.05 (0.16)	0.04 (0.34)	0.88 (0.44)
non-learners	0.17 (0.13)	0.17 (0.14)	0.16 (0.19)	0.49 (0.54)
Controls	0.08 (0.26)	0.13 (0.26)	0.12 (0.24)	0.67 (0.25)

### Parameter-level Inference

Having identified the fully connected model family as the most likely model architecture, we subsequently analyzed the model parameters resulting from BMA within that family. The 3×2×6 mixed ANOVA with between-subjects factors group (learners, non-learners, or controls), and within-subjects factors training run (first run, last run) and BMA parameter (V-SPL, SPL-V, up_V, up_SPL, up_V-SPL, up_SPL-V) revealed a significant main effect of group (F(1,2) = 5.49, p = 0.04), a significant main effect of BMA parameter (F(1,5) = 3.66, p = 0.01), and a strong trend towards significance in the 3-way interaction between group×training run×BMA parameter (F(1,10) = 1.91, p = 0.06). When replacing the last training run with the second to last training run (in order to avoid potential double dipping), there was no more main effect of group, no more main effect of BMA parameter, but the 3-way interaction between group×training run×BMA parameter was significant (F(1,10) = 2.44, p = 0.01).

Due to the strong 3-way interaction trend, and due to the predicted differential performance of the BMA parameters after compared to before training across the three groups, we performed the following tests in the 3 groups separately. A 2-way repeated measures ANOVA, with the factors training run and BMA parameter in the learners, revealed a significant main effect of BMA parameter (F(1,5) = 3.06, p = 0.02), and a significant interaction between the factors training run×BMA parameter (F(1,5) = 7.52, p<0.01). The interaction between the factors training run×BMA parameter was also significant when replacing the last training run with the second to last training run (in order to avoid potential double dipping; F(1,5) = 4.00, p<0.01).

**Figure 5:**
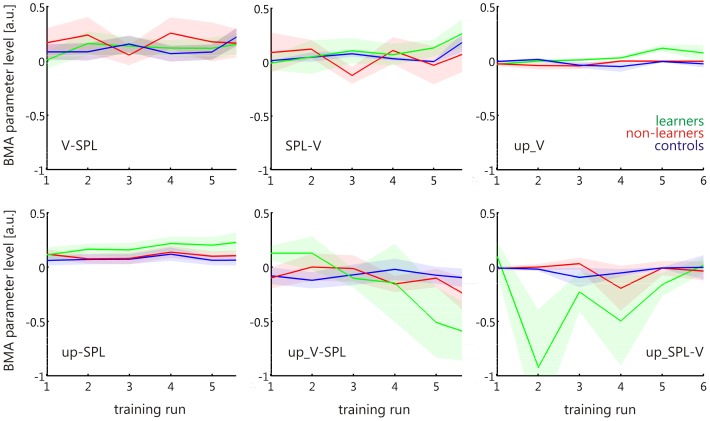
BMA parameter changes across neurofeedback training runs. While there were no significant changes in BMA parameter for the non-learners (red) and for the controls (blue), the learners (green) showed a significant increase in the top-down connection strength from cSPL to the visual ROI (SPL-V) that is independent of up-regulation. Further, the learners showed a significant increase in the effect of up-regulation on the visual ROI (up_V), and in the effect of up-regulation on the cSPL (up_SPL). The learners also showed a significantly decreasing effect of up-regulation on the bottom-up connection from the visual ROI to the cSPL (up_V-SPL). Shaded areas represent one standard error of the mean.

The same test in the non-learners revealed no significant main effect nor interaction (all ps >0.05), and the controls only showed a main effect of BMA parameter (F(1,5) = 2.91, p = 0.04; F(1,5) = 6.90, p<0.01 when replacing the last with the second to last training run). To better characterize connection strength increases or decreases across all 6 neurofeedback training runs in the learners, we plotted the BMA parameter changes over runs (Figure 5), and calculated linear regressions for each of them. We found a significant increase in the top-down connection strength from cSPL to the visual ROI (SPL-V, r^2^ = 0.77, F(1,4) = 13.11, p = 0.02; r^2^ = 0.77, F(1,3) = 9.83, p = 0.05 when replacing the last with the second to last training run) which is independent of the factor up-regulation. Further, we found a significant increase in the positive effect of up-regulation on the visual ROI (up_V, r^2^ = 0.78, F(1,4) = 14.34, p = 0.02; r^2^ = 0.85, F(1,3) = 17.46, p = 0.03 when replacing the last with the second to last training run) and on the cSPL (up_SPL, r^2^ = 0.88, F(1,4) = 29.13, p<0.01; r^2^ = 0.81, F(1,3) = 12.83, p = 0.04 when replacing the last with the second to last training run), and a significant decrease in the effect of up-regulation on the bottom-up connection from the visual ROI to the cSPL (up_V-SPL, r^2^ = 0.93, F(1,4) = 52.86, p<0.01; r^2^ = 0.88, F(1,3) = 21.46, p = 0.02 when replacing the last with the second to last training run). No significant changes were found for the non-learners and the controls (all ps >0).

## Discussion

We showed that neurofeedback training of visual cortex activity is associated with increased effective connectivity between the visual ROI and the cSPL. In the first neurofeedback training run, no specific model architecture dominated (Figure 4; Table 1). In contrast, in the last neurofeedback training run, the fully connected model family clearly dominated. Hence, the interaction between the visual ROI and the cSPL increased with neurofeedback training of visual cortex activity.

**Figure 6:**
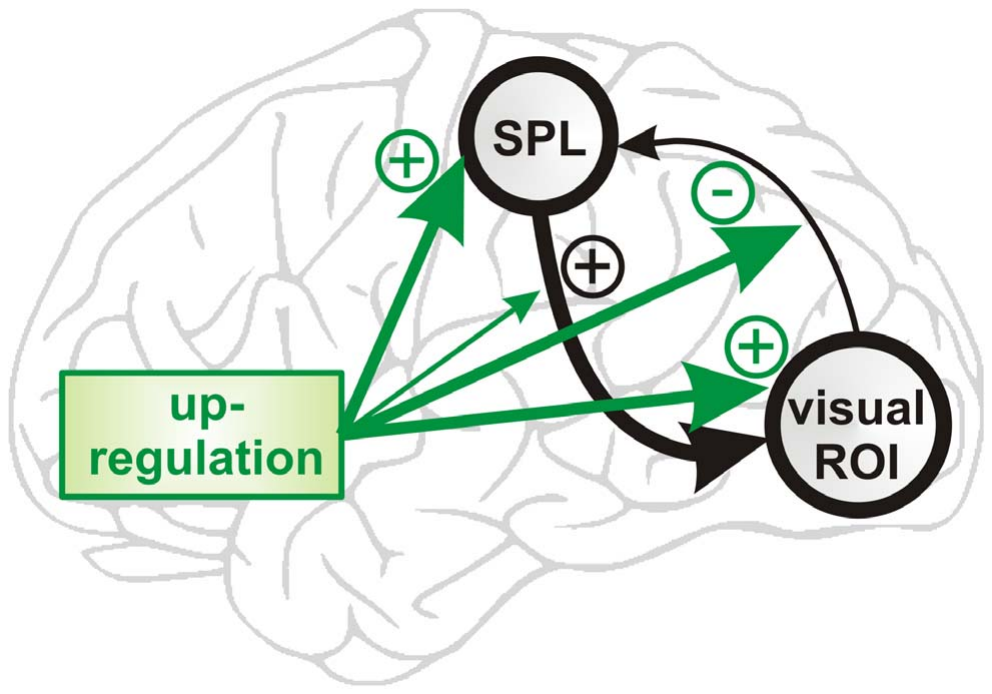
Connectivity changes underlying neurofeedback training. Successfully learning control over visual cortex activity was associated with increased top-down connectivity from cSPL onto the visual ROI. Further, up-regulation increasingly activated the visual ROI and the cSPL, but progressively reduced the bottom-up connection from the visual ROI onto the cSPL.

However, the increased dominance as training progressed of the fully connected model family was found in all experimental groups. It thus might reflect practicing self-regulation, but it cannot explain how learning visual cortex control is mediated, and why some participants learned self-regulation of visual cortex activity but others did not. Any neural substrate underlying successful learning of control over visual cortex activity must (a) show a systematic change across the neurofeedback training runs, and must (b) be specific to the learners (i.e. not be found in the non-learners or controls). When investigating the parameters of the fully connected model family, we found connectivity changes that fulfilled these two criteria (Figure 5). Specifically, we found that there was a systematic increase in top-down connectivity strength from the cSPL onto the visual ROI, which was only found in the learners. Also, the effect of up-regulation on the visual ROI and on the cSPL increased with training, and this only in the learners. Most pronounced and again only found in the learners, the effect of up-regulation on the bottom-up connectivity from the visual ROI onto the cSPL decreased significantly from positive to negative BMA parameter levels. This indicates that with training, the learners decreased the bottom-up connectivity from the visual ROI to the cSPL during up-regulation. Overall, these results suggest that learned control over visual cortex activity was mediated by increasingly effective top-down control, and by a reduction in bottom-up processing (Figure 6).

The connectivity pattern we found is in line with the use of visual-spatial attention and imagery, which was the cognitive control strategy that participants reported using (on debriefing) to control the neurofeedback signal [12]. Indeed, top-down control mechanisms such as attention and imagery can modulate visual cortex activity [40], [41], [42], [43], [44], [45], [46]. Moreover, the SPL is involved in directing covert visual-spatial attention [18], [19], [20], [21] and in cognitive control [19], [22], [23]. Finally, other recent DCM studies show that visual attention and imagery is associated with modulation of parietal cortex activity, and with strengthening of top-down connections from parietal to visual areas [26], [47], [48], [49], [50].

In our previous study, we did not find differences between participants who learned to control their visual cortex activity and those who did not with respect to introspective measures obtained during participant debriefing and psychological questionnaires. Learners as well as non-learners/controls used similar cognitive strategies, and showed the same attentional effort and vividness of visual imagery [12]. Our previous exploratory PPI analysis suggested however that control over the visual cortex was mediated by the interaction between the visual ROI and the cSPL. Our current DCM analysis confirms this hypothesis. It reveals that successful neurofeedback training of visual cortex activity involves specific parietal-visual network changes that may be closely linked to efficient deployment of top-down visual attention and imagery. The absence of such changes in effective connectivity in the non-learners and controls might explain their failure at learning to regulate visual cortex activity using neurofeedback.

Using neurofeedback to learn control over a brain ROI requires the recruitment of, and changes in, associated brain networks [14], [15], [16], [17]. In order to understand the neural underpinnings of successful neurofeedback learning, it will thus be important to identify the underlying network dynamics. Although the underlying network changes are specific to the trained ROI, characterizing these changes might allow the efficiency of future neurofeedback training studies targeting similar ROIs to be increased. Such analyses will potentially also help to identify brain networks that might be more efficiently trained using the recently developed real-time DCM neurofeedback approach, which allows to train brain networks directly [51]. Finally, identifying the connectivity changes associated with training a specific ROI might allow to evaluate the clinical relevance of the neurofeedback approach, especially for neuropsychiatric conditions which are associated with abnormal patterns of connectivity, such as hemispatial neglect [52], [53], depression [54], [55], and anxiety disorders [56], [57].
